# Social and Physical Environmental Characteristics Associated With Adult Current Cigarette Smoking

**DOI:** 10.5888/pcd16.180373

**Published:** 2019-06-06

**Authors:** Ralph S. Caraballo, Ketra L. Rice, Linda J. Neff, Bridgette E. Garrett

**Affiliations:** 1 National Center for Chronic Disease Prevention and Health Promotion, Office on Smoking and Health, Centers for Disease Control and Prevention, Atlanta, Georgia

## Abstract

**Introduction:**

Our objective was to identify social and physical environmental factors associated with current cigarette smoking among adults by metropolitan county in the United States.

**Methods:**

We linked cigarette smoking data from the 2012 Behavioral Risk Factor Surveillance System (BRFSS) Selected Metropolitan Area Risk Trends (SMART) data set to 7 social and physical environmental characteristics: county type (metropolitan designation), primary care physician density, income inequality, percentage of the population that was a racial/ethnic minority, violent crime rate, education, and percentage of county residents with low income and no health insurance, all obtained from several county data sets. Spatial regression and hierarchical logistic regression modeling were performed.

**Results:**

Results showed that metropolitan counties with a high proportion of non-Hispanic white adults (*P* < .001), lower education levels (high school graduate or less) (*P* < .001), and high violent crime rates (*P* < .001) had a higher adult cigarette smoking prevalence than other metropolitan counties. Spatial models showed 63.3% of the variability in county cigarette smoking prevalence was explained by these 3 factors as well as county type (based on population size of the of metropolitan area), primary care physician density, and percentage of county residents with low income and no health insurance. At an individual level, results showed that as the density (population) of primary care physicians increased in a county, the odds of being a current smoker decreased (OR, 0.980; *P* = .02).

**Conclusion:**

We found a significant association between adult cigarette smoking and county social and physical environmental factors. These place-based factors, especially social environmental characteristics, may reveal tobacco-related disparities to be considered when developing strategies to reduce tobacco use.

**Summary**

**What is already known on this topic?**

International studies show that place-based characteristics (social and physical environment) are associated with cigarette smoking. However, limited data are available about whether place-based characteristics are associated with cigarette smoking among US adults.

**What is added by this report?**

Of 7 metropolitan county characteristics studied, 3 (counties with a high proportion of non-Hispanic white adults, lower education levels, and high violent crime rates) had a higher smoking prevalence than their counterpart metropolitan counties.

**What are the implications for public health practice?**

Findings from this study highlight the need for more novel approaches, such as collaborations between tobacco use prevention and control organizations and crime prevention entities, when addressing tobacco-related disparities.

## Introduction

Although progress has been made in reducing cigarette smoking among the general population, cigarette smoking is still the leading cause of preventable disease and death in the United States (1). In addition, comprehensive tobacco control policies have contributed to reductions in cigarette smoking among the US population, but not all populations are universally covered by these policies, and disparities in cigarette smoking persist among population subgroups and geographic regions (1).

Current cigarette smoking is associated with individual characteristics, such as age, sex, race/ethnicity, income, education, and mental health status (2–4); however, less information is available about the association between cigarette smoking and social and physical environmental characteristics (ie, place characteristics) among communities. From a physical perspective, the concept of place examines the natural and built environments and how they affect health behaviors; from a social perspective, place examines the relational aspects of people within communities or the social characteristics of communities and how they can affect health behaviors (5). Determining the influence of place-based characteristics on cigarette smoking can help reduce tobacco-related disparities. Addressing place-based factors may further reduce the disparities in cigarette smoking, leading to improved health equality among all residents wherever they live (1).

Many international studies show that place-based characteristics are associated with cigarette smoking, including neighborhood deprivation, disadvantage, or crime (6–15); tobacco retail outlet density (16,17); limited opportunities for recreation (18); local population composition (ie, percentage of population that comprises racial/ethnic minorities) (19–21); and degree of urbanization (22,23). In addition, some data show that place-based characteristics, such as high concentrations of convenience stores, are linked to tobacco use in the United States, even after adjusting for several individual-level characteristics (24). However, limited data are available about whether place-based characteristics are associated with cigarette smoking among the US population. Thus, we explored the concept of place as those social and physical environmental characteristics of the geographic location where people live that might affect their cigarette smoking behavior.

The objectives of this study were to 1) assess the association between selected place-based characteristics and the prevalence of current cigarette smoking among US adults by county; and 2) determine the potential influence of selected place-based characteristics on cigarette smoking after controlling for known individual-level characteristics associated with cigarette smoking (ie, age, sex, race/ethnicity, education level, and health status).

## Methods

We conducted spatial analyses to examine the association between selected place-based metropolitan county characteristics and county-level cigarette smoking. Spatial analyses were used to examine aggregate-level patterns and associations in the data; however, aggregate associations can falsely attribute aggregate patterns to individual characteristics (objective 1). To diminish the potential for such bias, we further conducted hierarchical logistic regression to explore the variation in individual cigarette smoking status among counties by examining the individual characteristics at one level of analysis and the characteristics of the counties (objective 2). Both statistical methods were used to further explain the relationships that may occur at the aggregate level and to determine if the pattern of these relationships existed at both an individual and aggregate level.

### Data

Current cigarette smoking status was obtained from 182,172 survey respondents residing in 179 US metropolitan counties by using the 2012 Behavioral Risk Factor Surveillance System (BRFSS) Selected Metropolitan Area Risk Trends (SMART) data (25). BRFSS is an annual, state-based landline and cellular telephone survey among US adults aged 18 or older. The 2012 BRFSS SMART database consists of self-reported information (participants’ responses to the BRFSS survey) on current cigarette smoking in counties with 500 or more adult respondents. Current cigarette smokers were defined as respondents who reported smoking at least 100 cigarettes during their lifetime and who, at the time they participated in the survey, reported smoking “every day” or “some days.” These observations were used to estimate an aggregate measure of county-level cigarette smoking. County-level cigarette smoking prevalence was used as the outcome variable in spatial analyses. The individual smoking status of survey respondents was used as the outcome variable in hierarchical regression models. Additional individual-level variables from the BRFSS SMART, including age, sex, race/ethnicity, education, health insurance coverage, and health status, were also included in hierarchical modeling.

Aggregate county-level current smoking prevalence was calculated by using small area estimation methods. Data on county social and physical environmental influencers were obtained from several data sets (Appendix). For some of these data sets, data were not available for 2012, only for 2010 or 2013. We decided to use the data for 2010 or 2013, if available, because measures of place do not substantially change from one year to the next. Variables in analyses of the county physical environment were county type (metropolitan designation) and primary care physician (PCP) density. Variables in analyses of the county social environment were income inequality measured with the Gini index, which is a measure of statistical dispersion (26), percentage of minority population (Hispanic, non-Hispanic black, non-Hispanic Asian, non-Hispanic other), violent crime rate, education (high school diploma or less vs more than a high school education), and percentage of county residents with low income and no health insurance.

### Data analysis

We assessed prevalence of sociodemographic indicators of the individuals and of county-level characteristics. Correlation analysis was conducted with all selected county-level variables to determine if a high correlation between these variables existed. After finding that no pair of variables were strongly correlated (no correlations ≥0.8), a bivariate analysis was conducted to determine which of the variables were associated with current cigarette smoking (a Pearson correlation analysis was used if both variables were continuous and the Spearman correlation if a binary or ordinal variable). We then conducted spatial analyses to examine the relationship between county cigarette smoking and metropolitan county type, PCP density, percentage of minority population, violent crime rate, low-income uninsured, and education, variables that were found to have a bivariate association with smoking.

Spatial statistics were used to test for spatial autocorrelation. The Moran I statistic was used to measure the global spatial autocorrelation of the data to determine whether county observations were clustered. We then created local indicators of spatial association maps to visualize where local clustering existed and determine whether those locations were significant. The type of spatial autocorrelation was further examined by testing for spatial lag and spatial error. We conducted ordinary least squares regression to diagnose the presence of lag or error. The spatial lag model assumes that the prevalence of current cigarette smoking at one location (county) is affected by variables predicting cigarette smoking at nearby locations (counties). In contrast, the spatial error model assumes that cigarette smoking in one location (county) is not affected by predictor variables at nearby locations, but might be attributed to unmeasured predictor variables or the use of spatial data, regardless of the model being theoretically spatial or not. The robust Lagrange multiplier test indicates the presence of error and confirms that a spatial error regression model is needed to correct for autocorrelation. All aggregate-level analysis was conducted by using Stata version 14 (StataCorp LLC) and GeoDa version 1.10 (Center for Spatial Data Science, University of Chicago) software.

The final analysis conducted was a hierarchical logistic regression model to help explain what was observed in the spatial analyses and to identify the influence of both county-level and individual-level characteristics on the likelihood of being a current smoker. We created a 2-level model with the individual’s characteristics at the first level (level-1) and the social and physical environmental characteristics at the second level (level-2). Because current smoking in a county might vary on the basis of individual characteristics, we included respondents’ age, sex, race/ethnicity, education, health insurance coverage status, and self-reported health status. At level-2, we included variables reflecting the county type, PCP density, percentage of minority county population, low-income uninsured population, and violent crime rate.

We conducted 4 separate hierarchical models. Model 1 was an intercept only (null) model and did not contain any variables, but only incorporated county random effects to give us a sense of how much variability in current cigarette smoking existed within and between counties. Model 2 (random coefficients model) tested the relationship between each level-1 characteristic (age, sex, race/ethnicity, education, health insurance coverage, and self-reported health status) and the odds of being a current smoker. Model 3 (means as outcomes model) tested the significance and direction of the relationship between the county-level (level-2) variables and the odds of being a current smoker. Model 3 included only level-2 predictors, which helped provide the clearest evidence of which county-level factors in the model were contributing to the between-county variance in adult smoking. Last, model 4 (random intercepts and slopes model) built on the previous 2 models (models 2 and 3) by estimating the odds of being a current smoker with all level-1 and level-2 predictors accounted for simultaneously in the model. Model 4 also tested for specific interactions between the level-1 (respondents’ characteristics) and level-2 (county-level characteristics) variables. Hierarchical modeling was conducted by using HLM 7.02. (Scientific Software International). For results to qualify as significant as a whole, both levels had to be significant at *P* less than .05.

## Results

Table 1 provides unweighted descriptive statistics for the respondents’ characteristics and for aggregate county-level characteristics used in the analysis. Most respondents in the study were female (59.0%), non-Hispanic white (73.3%), and had health insurance coverage (89.0%). The highest proportion by age was adults aged 45 to 64 (39.2%); by education, some college or higher education (67.7%); and by self-reported health status, 33.5% reported to be in very good health. For county characteristics, large central metro (≥1 million population plus principal city), large fringe metro (≥1 million), and medium metro (250,000–999,999) had a similar proportion distribution in our study; small metro (populations <250,000) counties accounted for 15.1% of all studied counties. The mean percentage of minority residents was 33.6%, and about a third of adults with incomes less than 250% of the federal poverty guidelines living in these studied counties did not have health insurance. The mean PCP density was 6.6 per 10,000 population; mean income inequality index was 0.45 (ranged from 0 to 1.0, with 1.0 meaning perfect or total inequality); and the rate of violent crimes was 448.4 per 100,000 population.

**Table 1:** Descriptive Statistics (Percentage, Mean, or Rate) and 95% Confidence Intervals of 182,172 Survey Respondents and 179 Aggregated US Counties, Behavioral Risk Factor Surveillance System, 2012

Characteristic	Unweighted % (95% CI)
**Respondent Characteristics**	
**Current cigarette smoker^a^**	17.7 (17.0–18.5)
**Age, y**	
18–24	5.7 (5.7–5.9)
25–44	25.5 (25.0–25.9)
45–64	39.2 (38.8–39.8)
≥65	29.4 (28.9–30.0)
**Sex**	
Male	41.0 (40.8–41.2)
Female	59.0 (58.8–59.2)
**Race/ethnicity**	
Non-Hispanic white	73.3 (73.1–73.5)
Non-Hispanic black	10.4 (10.2–10.5)
Hispanic	9.1 (8.9–9.2)
Non-Hispanic Asian	3.1 (2.9–3.1)
Non-Hispanic other	4.1 (4.1–4.2)
**Highest attained education**	
High school graduate or equivalent or less	32.3 (32.1–32.5)
Some college or more	67.7 (67.5–67.9)
**Health insurance**	
Yes	89.0 (88.8–89.1)
No	11.0 (10.8–11.1)
**Self-reported health status**	
Excellent	19.5 (19.4–19.7)
Very good	33.5 (33.3–33.7)
Good	29.8 (29.6–30.0)
Fair	12.5 (12.4–12.6)
Poor	4.7 (4.6–4.8)
**County Characteristics**	
**Current cigarette smoking prevalence^b^**	18.3 (17.7–18.9)
**County type**	
Percentage of large central metro^c^	24.6 (18.8–31.5)
Percentage of large fringe metro^d^	29.6 (23.3–36.8)
Percentage of medium metro^e^	30.7 (24.4–37.9)
Percentage of small metro^f^	15.1 (10.5–21.2)
**Primary care provider density per 10,000 population^g^**	6.6 (6.3–7.1)
**Gini index, mean^h^**	0.45 (0.44–0.45)
**Percentage of minority population^i^**	33.6 (30.7–36.4)
**Violent crime per 100,000 population^j^**	448.4 (407.6–489.2)
**Percentage of low-income uninsured population^k^**	33.6 (30.7–36.4)
**Percentage with high school diploma or less**	38.8 (37.6–40.1)

a Self-reported current cigarette smokers are respondents who answered to have smoked 100 or more cigarettes (5 packs) in their life and who now smoke cigarettes every day or some days.

b County current cigarette smoking prevalence was calculated by using county-level small area estimation methods.

c Large central metro counties in metropolitan statistical areas (MSAs) of 1 million or more population that either contain the entire population of the largest principal city of the MSA, the largest principal city of the MSA, or at least 250,000 inhabitants of any principal city of the MSA.

d Large fringe metro counties in MSAs of 1 million or more population that did not qualify as large central metro counties.

e Medium metro counties in MSAs of populations of 250,000 to 999,999.

f Small metro counties in MSAs of populations less than 250,000.

g The number of primary care physicians in the county per 10,000 of the county population.

h The Gini index measures the degree of inequality in the distribution of family income in a country, region, or county. The more nearly equal a county’s income distribution, the lower its Gini index. The more unequal a county’s income distribution, the higher its Gini index. If income were distributed with perfect equality the index would be zero; if income were distributed with perfect inequality, the index would be 1.

i Percentage of people living in the county classified other than non-Hispanic white.

j The number of violent crimes committed in the county per 100,000 population.

k Those aged 18–64 years with low income and uninsured.

### Spatial analyses

In Table 2, the Moran I statistic of 0.288 (*P* < .001) indicated significant spatial autocorrelation in the data — counties and their associated characteristics tended to be clustered together. Results of the spatial error regression model showed that 63.3% of the variability in county cigarette smoking was explained by the 6 county place-based characteristics included in the model. Of these place-based variables measured, percentage of population that was racial/ethnic minority, violent crime rate, and education level were significant.

**Table 2:** Spatial Regression Models Measuring the Association Between 179 Aggregated US Metropolitan Counties and County Cigarette Smoking Prevalence, 2012

Characteristic	OLS^a^			Spatial Lag^b^^,^ c			Spatial Error^b^^,^ c ^,^ d		
	Coefficient	T-Statistic	*P* Value	Coefficient	Z-Value	*P* Value	Coefficient	Z-Value	*P* Value
ρ (WPrevalence)				0.015	0.579	.56			
λ							0.383	5.390	<.001
Constant	5.103	2.773	.006	4.783	2.510	.012	7.465	4.111	<.001
Metropolitan county type	0.305	0.233	.192	0.337	1.432	.15	0.187	0.818	.41
Primary care physician density	0.124	1.223	.22	0.126	1.270	.20	0.060	0.638	.52
Minority population	−0.146	−9.349	<.001	−0.146	−9.548	<.001	−0.143	−9.191	<.001
Violent crime rate	0.007	7.109	<.001	0.007	7.089	<.001	0.006	7.020	<.001
Low-income uninsured population	0.062	2.234	.026	0.064	2.325	.02	0.038	1.346	.18
Education	0.291	9.299	<.001	0.288	9.355	<.001	0.277	9.048	<.001
Adjusted *R* ^2^	0.562			0.563			0.633		
Log-likelihood	−437			−437			−426		
Akaike information criterion	888			889			866		
Moran I	0.288		<.001						
Robust Lagrange multiplier (lag)	0.624		.43						
Robust Lagrange multiplier (error)	20.574		<.001						
Likelihood ratio				0.347		.56	21.523		<.001

Abbreviations: OLS, ordinary least squares; ρ (WPrevalence), the coefficient in the spatial lag model (it measures the extent to which the dependent variable can be explained by the average of prevalence values of its nearest counties); λ, the coefficient in the spatial error model (lambda is also called the spatial error coefficient, and it will have a value of 0 if there is no spatial correlation between the error terms).

a OLS estimates provided as reference, with Moran I statistic denoting spatial autocorrelation.

b Maximum likelihood estimation.

c Modeled with rook contiguity spatial weights matrix.

d Spatial error results presented in text.

Forty-six states had at least 1 county represented in this study. Of 179 metropolitan counties in our study, 127 had borders with other counties in this study. Of these 127 counties, 28 were significant and positively correlated (*P* < .05) with similar high cigarette smoking prevalence as bordering counties; 3 counties were significant and negatively correlated with similar low smoking prevalence, whereas 96 counties that bordered some other studied counties were not spatially correlated.

### Hierarchical logistic regression analysis

The results of the hierarchical logistic regression analyses are presented in Table 3. In model 1, the influence of county on being an adult current smoker was significant (*P* < .001). The intra-class correlation coefficient showed that 7.4% of the variance in smoking status was attributable specifically to the within-county level. This variance component in the null model indicated the significance of county characteristics on smoking behavior.

**Table 3:** Odds of Being a Current Smoker, From Individual-Level and Metropolitan County–Level Characteristics, 2012^a^

Variable	Model 1 (Null Model)		Model 2 (Individual-Level Only)		Model 3 (County-Level Only)		Model 4 (Individual- and County-Level)	
	AOR (95% CI)	*P* Value	AOR (95% CI)	*P* Value	AOR (95% CI)	*P* Value	AOR (95% CI)	*P* Value
**Individual-level characteristics (n = 182,172)**								
Intercept	0.177 (0.170–0.186)	<.001	6.095 (5.333–6.966)	<.001			23.642 (20.731–26.963)	<.001
Health status (ordinal)			0.717 (0.706–0.728)	<.001			0.719 (0.708–0.730)	<.001
Has health insurance (vs no health insurance)			0.570 (0.543–0.598)	<.001			0.574 (0.553–0.597)	<.001
Age (continuous)			0.974 (0.973–0.976)	<.001			0.974 (0.974–0.975)	<.001
Education level (ordinal)			0.668 (0.654–0.681)	<.001			0.670 (0.658–0.683)	<.001
Male			1.195 (1.157–1.233)	<.001			1.195 (1.163–1.228)	<.001
Non-Hispanic black (vs non-Hispanic white)			0.900 (0.846–0.958)	<.001			0.900 (0.859–0.942)	<.001
Non-Hispanic Asian (vs non-Hispanic white)			0.437 (0.390–0.489)	<.001			0.438 (0.388–0.494)	<.001
Hispanic (vs non-Hispanic white)			0.380 (0.343–0.422)	<.001			0.384 (0.363–0.407)	<.001
All other non-Hispanic racial/ethnic minorities (vs non-Hispanic white)			1.173 (1.071–1.285)	<.001			1.171 (1.097–1.250)	<.001
**County-level characteristics (n = 179) **								
County type					1.034 (0.999–1.071)	.06		
Primary care physician density (continuous)					0.980 (0.964–0.997)	.02	0.992^b^ (0.986–0.998)	.011
Minority population (vs. non-Hispanic white) (continuous)					0.994 (0.992–0.997)	<.001	1.004^b^ (1.002–1.005)	<.001
Violent crime rate (continuous)					1.000 (1.000–1.001)	<.001		
Low-income uninsured population (continuous)					0.999 (0.996–1.004)	.98		
Variance of random effects	0.074							
χ^2^ (178 degrees of freedom)	1,675.987	<.001	10,699.90	<.001	1,185.859	<.001	10,704.96	<.001

Abbreviations: AOR, adjusted odds ratio; CI, confidence interval.

a Based on unit-specific model with robust standard errors, restricted penalized quasi-likelihood (PQL). All continuous variables grand mean centered. Model 1, intercept only model; model 2, random coefficients model; model 3, means as outcomes model; model 4, random intercepts and slopes model.

b Cross-level interactions: minority population and education; primary care physician density, and health.

In model 2, a random coefficients model with only individual participants’ variables (level-1) was performed with each level-1 predictor (age, sex, race/ethnicity, education, having health insurance, self-reported health status) being significant (*P* < .001). The odds of being a current smoker were lower for increasing age (OR, 0.974; *P* < .001), education level (OR, 0.668; *P* < .001), and self-reported good health (OR, 0.717; *P* < .001). Men had higher odds of being a current smoker than women (OR, 1.195; *P* < .001). Hispanic (OR, 0.380; *P* < .001), non-Hispanic black (OR, 0.900; *P* < .001), and non-Hispanic Asian (OR, 0.437; *P* < .001) adults had lower odds of being a current smoker than non-Hispanic white adults, whereas non-Hispanic other (OR, 1.173; *P* < .001) adults had higher odds of being a current smoker than non-Hispanic white adults. Those with health insurance coverage had lower odds of being a current smoker than those without health insurance (OR, 0.570; *P* < .001).

In model 3, the significance and direction of the relationship between the previously identified county-level (level-2) predictors and being a current smoker were assessed. The model showed that the PCP density and percentage of minority population of the county were significant with county-level smoking prevalence. The results showed the odds of being a current smoker were lower (OR, 0.980, *P* = .02) with increasing PCP density, as well as increasing percentage of minority population (OR, 0.994, *P* < .001). Neither the type of metropolitan county (*P* = .06) nor the proportion of low-income residents without health insurance (*P* = .98) were significantly associated with smoking status.

In model 4, the odds of being a current smoker were assessed when all significant residents’ characteristics (level-1) identified in model 2 and all significant metro county-level (level-2) factors identified in model 3 were simultaneously included in the model. The PCP density and minority population variables were added to the model simultaneously while maintaining the random effects of health and education. Results in model 4 showed a significant negative moderating effect of the county PCP density on the relationship between self-reported health status and smoking status (OR, 0.992, *P* < .011). This result suggests that a resident with a self-reported health status of good or better who lived in a county with high PCP density was less likely to be a current smoker than a resident with similar good or better self-reported health status who lived in a county with low PCP density.

## Discussion

Our study results fill a gap in the literature about the relationship between place and adult cigarette smoking in the United States. In the spatial models, we found that 63% of the variability in county-level current cigarette smoking was explained by metropolitan county type, PCP density, percentage of population that is racial/ethnic minority, violent crime rate, percentage of low-income uninsured, and education levels. Our findings are consistent with other studies of place-based influences on behavioral and health outcomes (5), which suggest that social environmental characteristics of residents (ie, education, crime, percentage population that is racial/ethnic minority) seem to explain most of the variability of outcomes, whereas physical environmental characteristics (ie, PCP density) explain a small percentage of the variability. Our study results also found significant place-based influences at the individual level by percentage of minority population and PCP density. Thus, our results suggest that place-based factors, especially social ones, are also important when considering the effect of current tobacco control interventions at the local level.

We found that cigarette smoking, at an aggregate level, increases as violent crime rates in metropolitan counties increase. Safe environments are conducive to community cohesion and foster neighborhood improvements and healthier behaviors; several studies found an association between high violent crime rates and high rates of adult cigarette smoking (8,27–29). Some authors attributed this association to high stressors caused by real or perceived unsafe environments (27,30). Even though factors that contribute to violence and unhealthy behaviors are complex, violence is preventable (31). To decrease the prevalence of cigarette smoking in counties, coordination within the public health sector and collaborations between the public health sector and crime prevention entities could be explored further.

Another key finding in this analysis is the moderating effect that PCP density has on the relationship between an individual’s self-reported health status and the likelihood of being a current cigarette smoker. We found that a resident with a self-reported health status of good or better who lives in a county with high PCP density is less likely to be a current smoker than a resident with a similar self-reported health status who lives in a county with low PCP density. A US study about the multiple benefits of PCP supply found that states with high PCP density per 10,000 population had lower cigarette smoking rates, less obesity, and higher seatbelt use than states with low PCP density (32). Primary care physicians are key influencers in tobacco control, both as advisers to quit smoking and as crucial societal leaders in the denormalization of tobacco use (33).

This study has some limitations. First, our study only included US metropolitan counties with sufficient responses (500 or more respondents); thus, assessed counties are not representative of all metropolitan counties in the United States. Most (62.8%) US counties are nonmetropolitan (34). Although our study included only US metropolitan counties, it captured areas where most people live. Second, for some place-based measures used, data were not available for 2012, only for 2010 or 2013. Third, variations in adult cigarette smoking between counties might be, in part, the result of differences in tobacco policies and strategies implemented at the county level. However, this information was not available at the county level. Finally, some respondents may have underreported their cigarette smoking (social desirability bias).

Findings from this study highlight the need for more novel approaches (eg, nontraditional partnerships) in tobacco use prevention and control that consider local-level, place-based factors when addressing tobacco-related disparities. Our analysis explains the role of selected social and physical environmental characteristics for more than half of the variability in county adult cigarette smoking prevalence. The analysis examined both residents (individual) and place-based (physical and social environmental characteristics) information to measure associations with cigarette smoking. To further understand how place-based characteristics influence cigarette smoking and mediate tobacco-related disparities, additional research at the micro (neighborhood) level is warranted. This type of information can help guide the placement of effective tobacco use prevention and control policies that consider the composition (ie, the person characteristics of those places) and the context of places to optimize the effectiveness of tobacco control strategies.

## Appendix County Variables Included in the Study, 2012

**Table:**

Characteristic	Database Used	Variable Definition
**Physical environment**		
County type	2013 National Center for Health Statistics Rural Urban County Codes, 2013	The metropolitan county categories are *Large central metro* counties in metropolitan statistical areas (MSAs) of 1 million or more population that either contain the entire population of the largest principal city of the MSA, the largest principal city of the MSA, or at least 250,000 inhabitants of any principal city of the MSA; *Large fringe metro* counties in MSAs of 1 million or more population that did not qualify as large central metro counties; *Medium metro* counties in MSAs of populations of 250,000 to 999,999; *Small metro* counties in MSAs of populations less than 250,000.
Primary care physician density	2012 Health Services and Resources Administration data, which has county health care professions files	The number of primary care physicians in the county per 10,000 of the county population.
**Social environment**		
Income inequality (Gini index)	2008–2012 American Community Survey 5-year estimates	The Gini index measures the degree of inequality in the distribution of family income in a country, region, or county. The more nearly equal a county’s income distribution, the lower its Gini index. The more unequal a county’s income distribution, the higher its Gini index. If income were distributed with perfect equality the index would be zero; if income were distributed with perfect inequality, the index would be 1.
Minority population	2008–2012 American Community Survey 5-year estimates	Percentage of people living in the county classified other than non-Hispanic white.
Violent crime rate	2010–2012 County Health Rankings, Uniform Crime Reporting, 2010–2012	The number of violent crimes committed in the county per 100,000 population.
Low-income uninsured population	US Census Bureau Small Area Health Insurance Estimates (SAHIE) for 2012	Those aged 18–64 years with low income and uninsured.
Education	2008–2012 American Community Survey 5-year estimates	Percentage of adult population in the county with a high school diploma or equivalenct or less.
